# Efficacy and safety of lipegfilgrastim versus pegfilgrastim: a randomized, multicenter, active-control phase 3 trial in patients with breast cancer receiving doxorubicin/docetaxel chemotherapy

**DOI:** 10.1186/1471-2407-13-386

**Published:** 2013-08-14

**Authors:** Igor Bondarenko, Oleg A Gladkov, Reiner Elsaesser, Anton Buchner, Peter Bias

**Affiliations:** 1 Dnipropetrovsk, State Medical Academy, 9, Dzerzhinsky Street, 49044, Dnipropetrovsk, Ukraine; 2 Chelyabinsk Regional Clinical Oncology Center, Chelyabinsk, Russia; 3 Teva Ratiopharm, Ulm, Germany

**Keywords:** Neutropenia, Febrile neutropenia, Breast cancer, Recombinant granulocyte-colony stimulating factor, Lipegfilgrastim, Pegfilgrastim

## Abstract

**Background:**

Lipegfilgrastim is a novel glyco-pegylated granulocyte-colony stimulating factor in development for neutropenia prophylaxis in cancer patients receiving chemotherapy. This phase III, double-blind, randomized, active-controlled, noninferiority trial compared the efficacy and safety of lipegfilgrastim versus pegfilgrastim in chemotherapy-naïve breast cancer patients receiving doxorubicin/docetaxel chemotherapy.

**Methods:**

Patients with high-risk stage II, III, or IV breast cancer and an absolute neutrophil count ≥1.5 × 10^9^ cells/L were randomized to a single 6-mg subcutaneous injection of lipegfilgrastim (n = 101) or pegfilgrastim (n = 101) on day 2 of each 21-day chemotherapy cycle (4 cycles maximum). The primary efficacy endpoint was the duration of severe neutropenia during cycle 1.

**Results:**

*Cycle 1:* The mean duration of severe neutropenia for the lipegfilgrastim and pegfilgrastim groups was 0.7 and 0.8 days, respectively (λ = −0.218 [95% confidence interval: –0.498%, 0.062%], p = 0.126), and no severe neutropenia was observed in 56% and 49% of patients in the lipegfilgrastim and pegfilgrastim groups, respectively. *All cycles:* In the efficacy population, febrile neutropenia occurred in three pegfilgrastim-treated patients (all in cycle 1) and zero lipegfilgrastim-treated patients. Drug-related adverse events in the safety population were reported in 28% and 26% of patients i006E the lipegfilgrastim and pegfilgrastim groups, respectively.

**Conclusion:**

This study demonstrates that lipegfilgrastim 6 mg is as effective as pegfilgrastim in reducing neutropenia in patients with breast cancer receiving myelosuppressive chemotherapy.

**Trial Registration:**

Eudra EEACTA200901599910

The study protocol, two global amendments (Nos. 1 and 2), informed consent documents, and other appropriate study-related documents were reviewed and approved by the Ministry of Health of Ukraine Central Ethics Committee and local independent ethics committees (IECs).

## Background

The efficacy of myelosuppressive chemotherapy regimens is often restricted by dose-limiting toxicities that can delay subsequent treatment cycles. One of the most common of these toxicities is a decrease in white blood cell counts, particularly of the neutrophil granulocytic lineage, clinically defined as neutropenia. Although neutropenia per se is asymptomatic, it is associated with many clinically important complications, including increased risk for opportunistic infection, febrile neutropenia (FN), sepsis, and related morbidity and mortality.

The risk of initial infection and subsequent complications is inversely proportional to the absolute neutrophil count (ANC) and begins to increase when the ANC is <1.5 × 10^9^/L. Consequently, the National Cancer Institute has defined neutropenia as an ANC < 1.0 × 10^9^/L [1]; this has also been designated as the minimum ANC required to initiate or continue chemotherapy cycles in many therapeutic clinical trials [2-4]. Because fever is often the only indication of an underlying infection in the setting of severe/febrile neutropenia, immediate hospitalization and administration of intravenous (i.v.) antibiotics is required [5,6]. According to the American Society of Clinical Oncology guidelines for antimicrobial prophylaxis and outpatient management of neutropenia in patients treated for cancer [7], patients with febrile neutropenia should receive initial doses of empirical antibacterial therapy within an hour of triage and should either be monitored for at least 4 hours to determine suitability for either outpatient management or admission to the hospital.

Recombinant granulocyte-colony stimulating factor (G-CSF) products have emerged as effective therapies for reducing the duration and incidence of chemotherapy-induced neutropenia and FN by stimulating neutrophil proliferation and differentiation in cancer patients [8,9]. Short-acting r-metHuG-CSFs (e.g., filgrastim) require daily subcutaneous (s.c.) injections during each chemotherapy cycle. The attachment of a polyethylene glycol (PEG) molecule (pegylation) to filgrastim (e.g., pegfilgrastim) decreases plasma clearance and extends the drug’s half-life in the body, allowing for less-frequent dosing [10,11]. Placebo-controlled clinical studies have shown significant reductions in the incidence of FN in patients treated with r-metHuG-CSF products [3,9]. Randomized, phase III, comparative studies have demonstrated similar trends in patients treated with once-per-cycle fixed-dose pegfilgrastim compared with once-daily filgrastim [2,12].

Lipegfilgrastim (XM22; Teva Pharmaceuticals Industries LTD, PetachTikva Israel) is a once-per-cycle, glyco-pegylated r-metHuG-CSF developed for the prevention of chemotherapy-induced neutropenia. Clinical data have shown lipegfilgrastim (6 mg) to be well tolerated in healthy volunteers, with dose-dependent increases in bioavailability and ANC comparable to that seen in pegfilgrastim (6 mg)-treated patients [13].

The primary objective of this study was to demonstrate the noninferiority of lipegfilgrastim compared with pegfilgrastim in patients with breast cancer during the first cycle of chemotherapy with respect to duration of severe neutropenia (DSN). Secondary evaluations of pharmacokinetic (PK) properties associated with lipegfilgrastim versus pegfilgrastim were also conducted in subsets of patients.

## Methods

### Study population

The study protocol was approved by the Ministry of Health of Ukraine Central Ethics Committee and all institutional review boards and local ethics committees of the participating centers. All patients gave written informed consent before any study-related procedures were performed. Twenty-seven centers in Russia and Ukraine enrolled and screened 218 breast cancer patients (stage II, III, or IV) from May 2010 to December 2010. Patients were eligible if they were at least 18 years of age, had no prior chemotherapy treatments and were eligible to receive four cycles of docetaxel and doxorubicin for the treatment of high-risk stage II, III, or IV breast cancer, had an Eastern Cooperative Oncology Group (ECOG) performance status ≥2, and had an ANC ≥1.5 × 10^9^/L and a platelet count ≥100 × 10^9^/L. Patients were required to have adequate cardiac function (including left ventricular ejection fraction ≥50% as assessed by echocardiography or equivalent method within 4 weeks prior to randomization), adequate hepatic function (i.e. alanine aminotransferase, aspartate aminotransferase both <2.5 × the upper limit of normal [ULN], alkaline phosphatase and bilirubin <5 × ULN). Patients were excluded if they had participated in a clinical trial 30 days before randomization; had previous exposure to filgrastim, pegfilgrastim, lenograstim, or other G-CSFs in clinical development less than 6 months prior to randomization; or had a known hypersensitivity to docetaxel or doxorubicin, filgrastim, pegfilgrastim, or lenograstim. Additional patient exclusion criteria included underlying neuropathy of grade 2 or higher, treatment with lithium at inclusion or during the study or antibiotics within 72 hours before chemotherapy, chronic use of oral corticosteroids, prior bone marrow or stem cell transplant, radiation therapy within 4 weeks prior to previous 5 years, and women who were pregnant or nursing.

### Study design

This study was a phase III, multinational, multicenter, randomized, double-blind, controlled study to evaluate the safety and efficacy of a fixed dose of lipegfilgrastim during the first cycle of chemotherapy versus pegfilgrastim in patients receiving a maximum of four cycles of combined myelosuppressive chemotherapy with doxorubicin 60 mg/m^2^ and docetaxel 75 mg/m^2^. Eligible patients were randomized in a 1:1 ratio to receive a single fixed-dose s.c. injection of either lipegfilgrastim 6 mg or pegfilgrastim 6 mg. Patients were assigned to treatment groups using a permuted block randomization with a block size of two, stratified by country. Chemotherapy was repeated every 3 weeks (unless a dose delay was necessary) for a maximum of four cycles.

### Treatment procedures

On day 1 of each chemotherapy cycle, patients received an i.v. bolus of doxorubicin (60 mg/m^2^) followed 1 hour later by a 1-hour i.v. infusion of docetaxel (75 mg/m^2^). Patients randomized to lipegfilgrastim or pegfilgrastim received a single s.c. injection of 6 mg of active study drug on day 2 of each cycle, approximately 24 hours after chemotherapy. Both the study drug and the active comparator drug were presented as identical prefilled syringes, and administration was performed via s.c. injection in the abdomen, upper arm, or thigh. Chemotherapy was repeated every 3 weeks for up to four cycles. Full-dose chemotherapy was started on day 1 of each cycle (day 22 of the previous cycle) only if a patient’s ANC was ≥1.5 × 10^9^/L and platelet count was ≥100 × 10^9^/L. A delay of up to 14 days in the initiation of the subsequent cycle was allowed to provide time for these hematologic parameters to be achieved.

### Schedule of assessments

Blood samples were collected for ANC determination within 24 hours of chemotherapy and then daily during cycle 1 up to day 15 or until an ANC of ≥2.0 × 10^9^/L was reached. Blood samples on day 2 were taken before study drug administration. ANC assessment for cycles 2, 3, and 4 was performed within 24 hours of chemotherapy and on days 1 and 3, then daily on days 5–15 of each cycle, until an ANC of ≥2.0 × 10^9^/L was achieved. Safety assessments (blood sampling for determination of antibodies, physical examinations, vital signs) were performed within 24 hours of chemotherapy in each cycle (day 1) and at the end of the study. Blood samples for PK assessments of lipegfilgrastim and pegfilgrastim were collected in a subset of patients during cycles 1 and 4. Patients recorded their oral body temperature twice daily until day 15 or until ANC reached ≥2.0 × 10^9^/L, and they were monitored for adverse events (AEs) and concomitant medication use throughout the study.

### Endpoints

The primary efficacy endpoint was the duration in days of severe neutropenia (grade 4, ANC <0.5 × 10^9^/L; Common Terminology Criteria for Adverse Events, version 3.0) in the first cycle of chemotherapy of the per-protocol (PP) study population. Incidence of FN in cycles 1–4 was a secondary efficacy endpoint and was defined as severe neutropenia in combination with one or more of the following: oral body temperature >38.5°C for at least 1 hour; documentation of neutropenic sepsis; and documentation of serious or life-threatening infection. Other secondary endpoints included duration of severe neutropenia in cycles 2–4; incidence and duration of severe and very severe neutropenia (ANC <0.1 × 10^9^/L) in cycles 1–4; and the lowest ANC level reached (ANC nadir) in each treatment cycle. Time to ANC nadir and to recovery (defined as a return of ANC to ≥2.0 × 10^9^/L) were also assessed, as was the incidence of i.v. antibiotic administration, hospitalization, and overall quality of life.

Standard PK parameters (including area under the curve [AUC], peak concentration [C_max_], and time to C_max_) were calculated from serum concentrations of lipegfilgrastim and pegfilgrastim measured at predefined time points in cycles 1 and 4.

Safety was assessed by the incidence of AEs using preferred terms designated by the Medical Dictionary for Regulatory Activities, changes in clinical chemistry, and changes in hematology laboratory values over time. Adverse events were classified as “bone-pain–related symptoms” with a comprehensive definition including the AE terms arthralgia, back pain, bone pain, neck pain, myalgia, and other musculoskeletal symptoms.

### Statistical analysis

The sample size of the study was based on a Poisson distribution of the target variable, DSN, as assessed by Monte-Carlo simulations. Allowing for a difference in DSN of 0.25 days in favor of pegfilgrastim in cycle 1, it was determined that a sample size of at least 86 patients per treatment group provided 90% power to reject the null hypothesis if the true DSN of lipegfilgrastim was within 1 day of that of pegfilgrastim.

Differences between treatment groups were analyzed using the two-sided 95% confidence interval (CI), calculated using a Poisson regression with identity link, including treatment, country, type of therapy (metastatic versus adjuvant), and body weight as fixed factors and with the last ANC value measured prior to the start of study treatment (baseline ANC) as a covariate. In both the intent-to-treat (ITT) and PP populations, lipegfilgrastim was to be considered noninferior to pegfilgrastim for the primary endpoint of DSN if, in cycle 1, the upper limit of the two-sided 95% CI for the difference in DSN was <1 day. The same variables were used in Poisson and logistic regression model estimates for the secondary efficacy endpoints.

For the calculation of PK parameters, concentration values below the lower limit of quantification (defined as 100 pg/mL) were set to 0 for t = 0 and set to missing for all other time points. For all time points used to calculate PK metrics, the actual time after G-CSF was used; if the actual time point was not available, the planned sample times were used.

### Analysis populations

The statistical analysis was based on separate, hierarchically organized analysis populations. Demographic data were analyzed for all of the following study populations: included not randomized (INR), ITT, PP, and safety populations. Efficacy data were analyzed for the ITT and PP populations. The safety endpoints were analyzed for the safety population. Demographic and baseline characteristics were also presented for the PK population.

The INR population comprised all patients enrolled but not randomized. The ITT population included all patients who were randomized to one of the study treatments at the baseline visit. The PP population was comprised of all patients in the ITT set for whom no major protocol violations occurred. The safety population included all randomized patients who received at least one dose or partial dose of study medication. The ITT and safety populations were identical, because all randomized patients were treated at least once with study medication. The PK sub-study population consisted of up to 20 patients per treatment group in selected centers. The centers and patients were chosen on the basis of logistic considerations.

The main population of interest for the efficacy measures was the PP population, because it is thought that assessing the effects of the agents in patients with no protocol violations may provide a more conservative representation of observed drug effects than the ITT population in clinical equivalence and noninferiority trials. Patients with major protocol violations included in ITT populations usually diminish possible treatment effect differences, i.e., patients with protocol violations usually act in favor of alternative hypotheses in statistical noninferiority or equivalence tests [14]. The ITT population statistics are included to affirm the results.

### Chemotherapy dose adjustments

The dose of docetaxel was reduced from 75 mg/m^2^ to 60 mg/m^2^ for patients who experienced severe and/or febrile neutropenia for more than 1 week, severe or cumulative cutaneous reactions, or severe (grade 3/4) peripheral neuropathy during therapy. The dose of doxorubicin was reduced from 60 mg/m^2^ to 45 mg/m^2^ for patients who experienced severe and/or febrile neutropenia for more than 1 week. The doses of both doxorubicin and docetaxel were reduced by 25% for subsequent cycles if patients had a platelet count of <2.0 × 10^10^/L at day 21 of a cycle.

Prohibited concomitant treatments included radiotherapy affecting the bone marrow, other investigational drugs or G-CSFs, transfusions of granulocytes, other cytotoxic treatment, and lithium. Prophylaxis with systemically (i.e., intramuscular, i.v., or oral) active antibiotics was not permitted except for patients at high risk for infection as assessed by the investigator. Treatment with antibiotics was allowed for any increased temperature >38.5°C (oral); if associated with severe neutropenia (ANC <0.5 × 10^9^/L); and for patients with a microbiologically, clinically, or radiologically documented infection. Antipyretics were only allowed if two consecutive temperature measurements >38.5°C (oral) at least 1 hour apart from one another were documented, or if fever occurred after treatment with systemic antibiotics had been started. Analgesics were to be avoided except in patients who experienced pain associated with chemotherapy or study drug. Corticosteroids (oral or i.v.) could be given if deemed necessary (e.g. to prevent or immediately treat a hypersensitivity reaction to a chemotherapeutic drug). Herceptin was not allowed during chemotherapy treatment but could be given at the end of the study visits (day 85).

## Results

### Patient disposition

**Figure 1:**
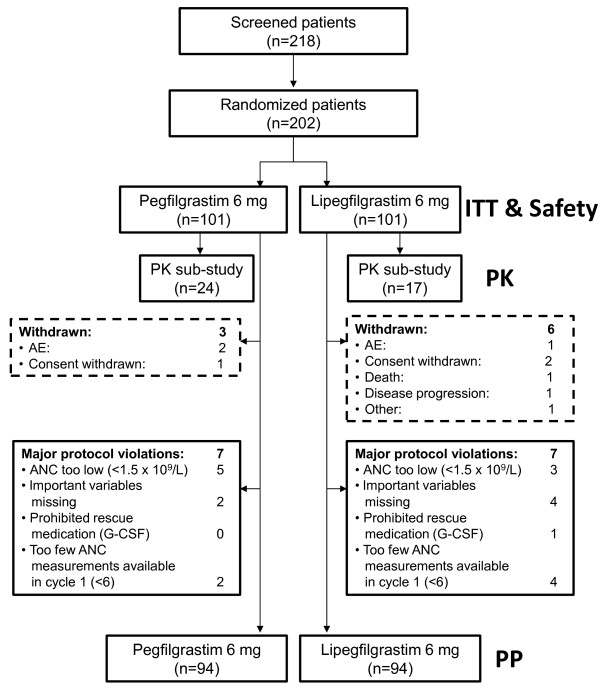
**Disposition of patients.** AE = adverse event, ANC = absolute neutrophil count.

Two hundred eighteen patients were screened for entry into the study. As shown in Figure 1, 202 patients were randomized (lipegfilgrastim: n = 101 and pegfilgrastim: n = 101), received at least one dose of active treatment and constituted both the efficacy ITT population and safety population. A total of 193 (95.5%) patients initiated all four cycles of chemotherapy. Seven patients with major protocol violations were excluded from the PP population in each treatment group (n = 94 per treatment group). The PK substudy population was composed of 41 patients (lipegfilgrastim: n = 17 and pegfilgrastim: n = 24) from the ITT population. The majority of patients in both treatment groups received chemotherapy as scheduled, with the mean percentage of doxorubicin and docetaxel actually administered reaching more than 98% in each group in each cycle. Thirty one patients in the lipegfilgrastim group and 36 patients in the pegfilgrastim group received delayed chemotherapy treatment in cycles 2–4. There were no dose omissions or reductions in the lipegfilgrastim group and eight in the pegfilgrastim group in cycles 2–4.

### Baseline characteristics

All patients in the ITT population were white, chemotherapy-naïve women. Overall, 39%, 48%, and 14% of patients in the lipegfilgrastim group and 36%, 45%, and 20% in the pegfilgrastim group had stage II, III, and IV breast cancer, respectively. Their mean ages ± standard deviation (SD) were 49.9 ± 10.1 years and 51.1 ± 9.4 years for the lipegfilgrastim and pegfilgrastim groups, respectively. The two groups were generally well balanced for other demographic factors and disease status at baseline (Table 1). Overall, 13 patients in the pegfilgrastim group and 15 patients in the lipegfilgrastim group had received previous radiotherapy. The majority of patients were receiving chemotherapy as adjuvant therapy in the lipegfilgrastim and pegfilgrastim groups (74.3% and 73.3%, respectively). Twenty-six percent of patients in the lipegfilgrastim group and 27% of patients in the pegfilgrastim group were receiving chemotherapy for treatment of metastatic disease. All baseline characteristics of the ITT population were similar to those of the PP population.

**Table 1:** Demographic and clinical characteristics

**Characteristic**	**Pegfilgrastim 6 mg (n = 101)**		**Lipegfilgrastim 6 mg (*n*** **=** **101)**	
	**Intent-to-treat/safety population**	**Per-protocol population**	**Intent-to-treat/safety population**	**Per-protocol population**
Mean age, years (± SD)	51.1 (9.4)	50.9 (9.3)	49.9 (10.1)	49.3 (10.0)
Age ≥65 years	7 (6.9)	6 (6.4)	7 (6.9)	6 (6.4)
Mean weight, kg (± SD) ≤	73.2 (14.6)	73.6 (14.8)	73.9 (17.1)	73 (16.4)
Gender, n (%)				
Female	101 (100.0)	94 (100.0)	101 (100.0)	94 (100.0)
Male	0 (0.0)	0 (0.0)	0 (0.0)	0 (0.0)
Country, n (%)				
Russia	63 (62.4)	61 (64.9)	63 (62.4)	60 (63.8)
Ukraine	38 (37.6)	33 (35.1)	38 (37.6)	34 (36.2)
Reason for chemotherapy, n (%)				
Adjuvant therapy	74 (73.3)	70 (74.5)	75 (74.3)	71 (75.5)
Treatment for metastatic disease	27 (26.7)	24 (25.5)	26 (25.7)	23 (24.5)
Disease stage, n (%)				
High-risk stage II	36 (35.6)	35 (37.2)	39 (38.6)	38 (40.4)
Stage III	45 (44.6)	44(46.8)	48 (47.5)	46 (48.9)
Stage IV	20 (19.8)	15 (16.0)	14 (13.9)	10 (10.6)
ECOG performance status,^*^ n (%)				
01	47 (46.5)	46 (48.9)	45 (44.6)	44 (46.8)
02	54 (53.5)	48 (51.1)	56 (55.4)	50 (53.2)
03	0 (0.0)	0 (0.0)	0 (0.0)	0 (0.0)
Months since first diagnosis				
Mean ± SD	6.1 ± 26.6	4.5 ± 20.3	5.3 ± 16.7	4.4 ± 15.6
Median (range)	1.0 (0–185.0)	1.0 (0–185.0)	2.0 (0–130.0)	1.0 (0–130.0)
Breast surgery, n (%)				
Yes	41 (40.6)	57 (60.6)	51 (50.5)	45 (47.9)
No	60 (59.4)	37 (39.4)	50 (49.5)	49 (52.1)
Months since last surgery				
N with surgery	60	57	50	45
Mean ± SD	9.1 ± 34.1	6.3 ± 25.8	6.7 ± 15.5	4.9 ± 12.3
Median (range)	1.0 (0–185.0)	1.0 (0–185)	1.0 (0–72.0)	1.0 (0–185)
Type of breast surgery,^†^ n (%)				
Breast-conserving surgery	7 (6.9)	6 (6.4)	4 (4.0)	2 (2.1)
Mastectomy	55 (54.5)	52 (55.3)	46 (45.5)	43 (45.7)
Axillary lymphadenectomy	46 (45.5)	44 (46.8)	44 (43.6)	40 (42.6)

^*^ECOG performance status: 0 = fully active, able to carry on all pre-disease performance without restriction; 1 = restricted in physically strenuous activity but ambulatory and able to carry out work of a light or sedentary nature, e.g., light housework, office work; 2 = ambulatory and capable of all self-care but unable to carry out any work activities; up and about more than 50% of waking hours.

^†^Patients could be counted in multiple categories.

*ECOG* Eastern Cooperative Oncology Group, *SD* standard deviation.

### Efficacy

#### Duration of severe neutropenia in cycle 1

In the PP population, the mean (±SD) duration of severe neutropenia, the primary efficacy endpoint, was comparable in both treatment groups: 0.8 ± 0.9 days in the active control pegfilgrastim group and 0.7 ± 0.9 days in the lipegfilgrastim group (Table 2). As a result, the study met its primary endpoint and lipegfilgrastim was noninferior to pegfilgrastim, with a 95% two-sided CI of −0.498%, 0.062% days (p = 0.1260). Results for the primary endpoint were similar in the ITT population with a mean DSN of 0.7 ± 1.0 for the lipegfilgrastim group and 0.9 ± 0.9 for the pegfilgrastim group (p = 0.1841).

**Table 2:** **Duration of severe neutropenia in cycles 1**–**4** (**per-protocol population**)

	**Cycle 1 (primary endpoint)**		**Cycle 2**		**Cycle 3**		**Cycle 4**	
	**Pegfilgrastim 6 mg**	**Lipegfilgrastim 6 mg**	**Pegfilgrastim 6 mg**	**Lipegfilgrastim 6 mg**	**Pegfilgrastim 6 mg**	**Lipegfilgrastim 6 mg**	**Pegfilgrastim 6 mg**	**Lipegfilgrastim 6 mg**
Mean ± SD	0.8 ± 0.9	0.7 ± 0.9	0.3 ± 0.6	0.1 ± 0.5	0.2 ± 0.4	0.1 ± 0.3	0.2 ± 0.5	0.2 ± 0.6
Median (range)	1 (0.0, 4.0)	0 (0.0, 4.0)	0 (0.0, 3.0)	0 (0.0, 3.0)	0 (0.0, 2.0)	0 (0.0, 2.0)	0 (0.0, 3.0)	0 (0.0, 3.0)
LS Mean (95% CI)^*^	−0.218 (−0.498%, 0.062%)		−0.123 (−0.282%, 0.036%)		−0.029 (−0.145%, 0.087%)		0.008 (−0.147%, 0.163%)	
*p* value^†^	0.1260		0.1287		0.6227		0.922	

^*^Least-squares mean comparison of lipegfilgrastim versus pegfilgrastim by Poisson regression with treatment, country, type of therapy, and weight class as class variables and absolute neutrophil count baseline as a covariable. Poisson regression with possible overdispersion.

^†^*p* value is based on null hypothesis of equality.

*CI* confidence interval, *LS* least-squares, *SD* standard deviation.

#### Incidence of febrile neutropenia in cycles 1 to 4

In the PP population, three patients (3.2%) who received pegfilgrastim and none who received lipegfilgrastim developed FN (according to the strict definition) during cycle 1. In the ITT population, three patients in the pegfilgrastim group (same patients as in the PP population) and one patient in the lipegfilgrastim group developed FN during the study. There was no statistically significant observed difference in the incidence of FN between the treatment groups where calculable. The patient taking lipegfilgrastim who was assessed by an investigator as having FN was excluded from the PP population due to major protocol violations, and there were insufficient data to confirm whether the patient had or did not have FN according to the strict definition.

#### Duration of severe neutropenia in cycles 2 to 4

The DSN in each cycle was comparable between the treatment and active control groups, with no observed statistically significant differences (Table 2). The mean DSN was consistently shorter in cycles 2–4 than in cycle 1 in both treatment groups. Of note, in each of cycles 2–4, more than 75% of the patients in each treatment group experienced no severe neutropenia. The results in the ITT population were consistent with those in the PP population.

#### Incidence of severe neutropenia in cycles 1 to 4

In the PP population, the incidence of severe neutropenia was not statistically significantly different between the treatment and active control groups during cycles 1, 3, and 4. In cycle 2, 21.5% of patients in the pegfilgrastim group and 8.5% of patients in the lipegfilgrastim group had severe neutropenia (p = 0.0130, Table 3). Most cases of severe neutropenia occurred in the first cycle, with 51.1% of patients in the pegfilgrastim cohort and 43.6% of patients in the lipegfilgrastim cohort having severe neutropenia during cycle 1 (p = 0.3409). The results of the ITT population were consistent with those of the PP population.

**Table 3:** **Incidence of severe neutropenia in cycles 1**–**4** (**per-protocol population**)^*^

	**Cycle 1**		**Cycle 2**		**Cycle 3**		**Cycle 4**		**Across all cycles**	
	**Pegfilgrastim 6 mg**	**Lipegfilgrastim 6 mg**	**Pegfilgrastim 6 mg**	**Lipegfilgrastim 6 mg**	**Pegfilgrastim 6 mg**	**Lipegfilgrastim 6 mg**	**Pegfilgrastim 6 mg**	**Lipegfilgrastim 6 mg**	**Pegfilgrastim 6 mg**	**Lipegfilgrastim 6 mg**
n/N	48/94	41/94	20/93	8/94	11/91	8/93	11/91	11/90	55/94	47/94
(%)	(51.1)	(43.6)	(21.5)	(8.5)	(12.1)	(8.6)	(12.1)	(12.2)	(58.5)	(50.0)
OR (95% CI)	0.745 (0.405%, 1.369%)		0.291 (0.110%, 0.769%)		0.676 (0.249%, 1.835%)		0.997 (0.391%, 2.545%)		0.708 (0.383%, 1.309%)	
*p* value^†^	0.3409		0.0130		0.4404		0.9958		0.2695	

^*^Multiple mentions per patient are possible.

^†^Pegfilgrastim 6 mg vs. lipegfilgrastim 6 mg (*p* values are based on a null hypothesis of OR = 1).

*CI* confidence interval, *OR* odds ratio.

#### Incidence and duration of very severe neutropenia in cycles 1 to 4

The incidence of very severe neutropenia over all cycles in the PP population was low in both groups (11.7% of pegfilgrastim patients and 6.4% of lipegfilgrastim patients; p = 0.2066). Similarly, the duration of very severe neutropenia in each cycle was short in both treatment groups, with no significant differences observed. The results in the ITT population were consistent with those in the PP population.

#### Absolute neutrophil counts

**Figure 2:**
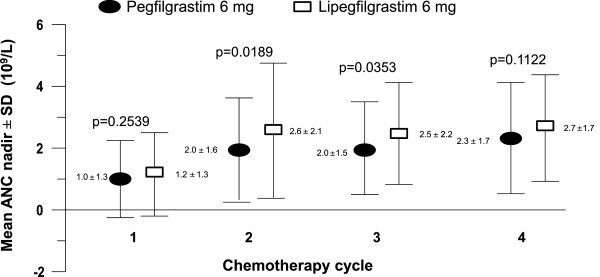
Absolute neutrophil count (ANC) nadir in cycles 1-4 (per-protocol population).

The depth of ANC nadir for each cycle was defined as the minimal ANC value for a patient in each respective cycle. The depth of ANC nadir in both PP population treatment groups was lowest in cycle 1, then increased to ≥2.0 × 10^9^/L in cycles 2–4. The depth of ANC nadir in cycle 1 was comparable in both treatment groups (*p* = 0.2539). In cycles 2, 3, and 4, the mean depth of ANC nadir had higher absolute values for patients treated with lipegfilgrastim compared with those treated with pegfilgrastim (2.6 vs. 2.0, 2.5 vs. 2.0, and 2.7 vs. 2.3 × 10^9^/L; p = 0.0189, p = 0.0353, and p = 0.1122, respectively; Figure 2).

**Figure 3:**
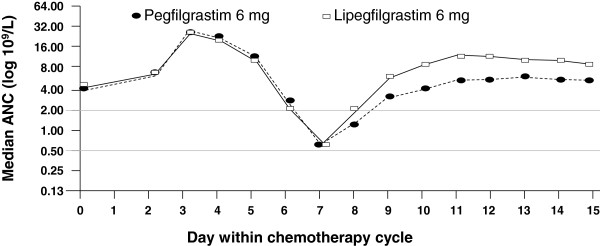
Median absolute neutrophil count (ANC) by day in chemotherapy cycle 1 (per-protocol population).

The median time of recovery to an ANC >2.0 × 10^9^/L in both PP population treatment groups was highest in cycle 1 (7 days for lipegfilgrastim, 8 days for pegfilgrastim; Figure 3). In cycles 1, 2, and 3, the time to ANC recovery was shorter for lipegfilgrastim-treated patients than for pegfilgrastim-treated patients (p < 0.05; Table 4). In cycle 4, the time to ANC recovery was comparable in both treatment groups.

**Table 4:** **Time to ANC recovery in cycles 1**–**4** (**per protocol population**)

	**Cycle 1**		**Cycle 2**		**Cycle 3**		**Cycle 4**	
	**Pegfilgrastim 6 mg**	**Lipegfilgrastim 6 mg**	**Pegfilgrastim 6 mg**	**Lipegfilgrastim 6 mg**	**Pegfilgrastim 6 mg**	**Lipegfilgrastim 6 mg**	**Pegfilgrastim 6 mg**	**Lipegfilgrastim 6 mg**
Mean ± SD	7.4 ± 3.6	5.9 ± 3.4	5.3 ± 4.6	3.6 ± 4.1	5.1 ± 4.3	3.9 ± 4.8	4.3 ± 4.7	3.3 ± 4.1
Median (range)	8 (0–21.0)	7 (0–12.0)	7 (0–18.0)	0 (0–15.0)	7 (0–13.0)	0 (0–21.0)	6 (0–21.0)	0 (0–13.0)
LS mean	−1.589		−1.661		−1.344		−0.802	
(95% CI)^*^	(−2.615%, –0.563%)		(−2.885%, –0.436%)		(−2.579%, −0.108%)		(−2.098%, 0.493%)	
*p* value^†^	0.0026		0.0082		0.0332		0.2234	

^*^Least-squares mean, 95% CI, and *p* value are for the Poisson regression analysis lipegfilgrastim – pegfilgrastim.

^†^*p* value is based on null hypothesis of equality.

*CI* confidence interval, *LS* least-squares, *SD* standard deviation.

#### Safety

The safety profile of lipegfilgrastim was similar to that of pegfilgrastim, with no patterns or trends indicative of increased lipegfilgrastim toxicity. Treatment-emergent adverse events (TEAEs) are summarized in Table 5. Most TEAEs reported were attributable to complications of myelosuppressive chemotherapy or the primary disease (i.e., alopecia, nausea, asthenia, neutropenia) and occurred in similar percentages of patients in each group during the study. The overall frequencies of almost all TEAEs decreased over chemotherapy cycles, with the highest frequencies in cycle 1 and lower frequencies in cycles 2–4.

**Table 5:** **Frequencies of treatment**-**emergent adverse events** (**TEAEs**)* (**safety population**)

**Category of TEAE**	**Pegfilgrastim 6 mg (N = 101)**	**Lipegfilgrastim 6 mg (N = 101)**
	**n (%)**	**n (%)**
Any TEAE	99 (98.0)	100 (99.0)
Drug-related TEAE = TEADR	26 (25.7)	28 (27.7)
Serious TEAE	7 (6.9)	3 (3.0)
Serious TEADR	1 (1.0)	1 (1.0)
Severe TEAE	35 (34.7)	26 (25.7)
Severe TEADR	2 (2.0)	1 (1.0)
Discontinued due to TEAE	2 (2.0)	3 (3.0)
Discontinued due to TEADR	1 (1.0)	0 (0)
Death	0 (0)	1 (1.0)

^*^Patients could be counted in multiple categories.

*TEAE* treatment-emergent adverse event; *TEADR* treatment-emergent adverse drug reaction.

Serious TEAEs were reported in seven (6.9%) pegfilgrastim patients and in three (3.0%) lipegfilgrastim patients. Three cases of FN were reported in the pegfilgrastim group and one was reported in the lipegfilgrastim group of the safety population. Severe TEAEs were reported in 34.7% of pegfilgrastim patients and 25.7% of lipegfilgrastim patients. One patient treated with a single dose of lipegfilgrastim died during this study. Upon autopsy, enterocolitis was proven as the cause of death and was determined not to be related to study medication by the investigator.

#### Adverse events of special interest

“Bone-pain–related symptoms” according to the comprehensive definition were the most commonly reported AE in 17 (16.8%) pegfilgrastim patients and in 24 (23.8%) lipegfilgrastim patients, but the difference was not statistically significant. Rates of component AEs such as bone pain (9.9% of pegfilgrastim patients and 13.9% of lipegfilgrastim patients), myalgia (5.9%, 8.9%), and arthralgia (2.0%, 5.0%) were also comparable between arms. None of the AEs related to bone-pain–related symptoms led to the discontinuation of study participation, and none were serious. All were mild or moderate in severity as expected under G-CSF treatment and were either well managed using standard analgesics or required no additional treatment.

#### Incidence of hospitalization and antibiotic treatment

Two patients in the pegfilgrastim group and one patient in the lipegfilgrastim group were hospitalized due to FN or infection. All three patients were hospitalized during cycle 1 (one pegfilgrastim patient for 6 days and the other for 5 days; the lipegfilgrastim patient for 1 day) and received antibiotics; the lipegfilgrastim patient also received antipyretics. One other patient in the pegfilgrastim group required antibiotics due to FN in cycle 1 but was not hospitalized. The lipegfilgrastim patient who was hospitalized due to FN was not included in the PP population because of protocol violations.

#### Pharmacokinetic subanalysis

The PK of 6 mg lipegfilgrastim and of 6 mg pegfilgrastim after s.c. administration were similar in many respects, but differed in the AUC. In cycle 1, descriptively, the geometric means of AUC_0-last_ and AUC_0-∞_ were higher for lipegfilgrastim compared with pegfilgrastim (14,157 ng/mL/h vs. 10,532 ng/mL/h and 14,184 ng/mL/h vs. 10,554 ng/mL/h, respectively). In cycle 4, the geometric means of AUC_0-last_ and AUC_0-∞_ were higher for pegfilgrastim (4812 ng/mL/h and 4839 ng/mL/h, respectively) compared with lipegfilgrastim (2975 ng/mL/h and 3588 ng/mL/h, respectively) but in both cases were lower than in cycle 1.

## Discussion

The results of this study demonstrate the noninferiority of lipegfilgrastim versus the active control pegfilgrastim in patients with breast cancer who are receiving myelosuppressive chemotherapy. The incidence and duration of severe neutropenia in patients who received lipegfilgrastim was similar to or lower than that of patients who received pegfilgrastim. The results of this study substantiate the findings of a previous lipegfilgrastim dose-finding study in breast cancer patients, which reported a mean DSN of 0.8 days in the 6-mg lipegfilgrastim group and 0.9 days in the 6-mg pegfilgrastim group in cycle 1 [15].

The results for the analyses of all secondary efficacy endpoints were consistent with those of the primary endpoint, with lipegfilgrastim demonstrating comparable efficacy to the active comparator pegfilgrastim. Where differences between the groups were observed, the differences favored greater antineutropenic activity for lipegfilgrastim compared with pegfilgrastim.

The DSN observed in either treatment group was considerably shorter than values reported in previous clinical studies of breast cancer patients who received similar myelosuppressive chemotherapy but were not treated with a G-CSF [4]. delGiglio et al. [4] reported a mean DSN of 3.8 days in cycle 1 in patients with breast cancer not receiving G-CSF support. A prolonged mean DSN of 5.7 days was also reported in a study of patients with non–small-cell lung cancer [16]. Moreover, historical data from breast cancer patients treated with pegfilgrastim reported a considerably longer mean DSN in cycle 1 of 1.8 days, compared with 0.7 days in lipegfilgrastim-treated patients in the current study [2].

There was a relatively low occurrence of FN in the present study. This is in contrast to a previous clinical study in breast cancer patients that applied the same chemotherapy regimen and had very similar inclusion and exclusion criteria in which a higher incidence of FN in pegfilgrastim-treated patients (9% [7 of 80 patients]) was reported [2]. This discrepancy was most likely the result of a less restrictive definition of FN (38.2°C measured once instead of 38.5°C measured twice with a 1-hour interval). The reductions in the rate of FN are in line with the results of a meta-analysis of 15 randomized, placebo-controlled trials in which rates of FN were significantly lower in G-CSF–treated groups than controls (22.4% vs. 39.5%, respectively [relative risk = 0.54; 95% CI: 0.43%, 0.67%; p < 0.0001]) [17].

Lipegfilgrastim had a favorable safety profile consistent with that of a G-CSF [10]. Frequencies of AEs were generally comparable between the treatment groups. The most commonly experienced AEs were alopecia, nausea, asthenia, neutropenia, bone pain, erythema, leukopenia, and diarrhea. The only AEs that differed in frequency by ≥5% between the treatment groups were alopecia, nausea, neutropenia, and vomiting. However, these differences were not considered to be clinically relevant, as all of the aforementioned AEs are known to be associated with the chemotherapy regimen or the underlying disease. G-CSF bone pain–related symptoms were comparable between treatment arms [11]. These side effects were mild or moderate in severity, well managed using standard analgesics, and did not lead to early discontinuation of study treatment.

The PK of lipegfilgrastim 6 mg and pegfilgrastim 6 mg after s.c. administration were generally similar with one key difference: lipegfilgrastim had a higher AUC_0-last_ and AUC_0-∞_ compared with pegfilgrastim in cycle 1. Given that both AUC parameters were almost 50% higher for lipegfilgrastim compared with pegfilgrastim in cycle 1, the activity of a 6-mg lipegfilgrastim dose would be expected to be greater than that of pegfilgrastim 6 mg. This is consistent with the efficacy and safety results observed in this study, with a trend toward a somewhat higher effect observed for lipegfilgrastim in terms of anti-neutropenic activity.

## Conclusions

The current study demonstrates that a single fixed-dose injection of lipegfilgrastim 6 mg was noninferior to the active control pegfilgrastim in patients with breast cancer receiving myelosuppressive chemotherapy. Overall, lipegfilgrastim has a safety profile that is consistent with a G-CSF and acceptable for the intended patient population.

## Abbreviations

AE: Adverse event; ANC: Absolute neutrophil count; AUC: Area under the curve; CI: Confidence interval; Cmax: Peak concentration; DSN: Duration of severe neutropenia; ECOG: Eastern Cooperative Oncology Group; FN: Febrile neutropenia; G-CSF: Granulocyte-colony stimulating factor; INR: Included not randomized; ITT: Intent-to-treat; i.v.: Intravenous; PEG: Polyethylene glycol; PK: Pharmacokinetic; PP: Per-protocol; s.c.: Subcutaneous; SD: Standard deviation; ULN: Upper limit of normal.

## Competing interests

Igor Bondarenko, MD, PhD, and Oleg A. Gladkov, MD, have no conflicts of interest to disclose. Reiner Elsaesser, MSc, Anton Buchner, PhD, and Peter Bias, MD, are employees of Teva Pharmaceuticals.

## Authors’ contributions

All authors read and approved the final manuscript.

## Pre-publication history

The pre-publication history for this paper can be accessed here:

http://www.biomedcentral.com/1471-2407/13/386/prepub
